# Canonical germinant receptor is dispensable for spore germination in *Clostridium botulinum* group II strain NCTC 11219

**DOI:** 10.1038/s41598-017-15839-y

**Published:** 2017-11-13

**Authors:** Charlien Clauwers, Cédric Lood, Bram Van den Bergh, Vera van Noort, Chris W. Michiels

**Affiliations:** 1 Laboratory of Food Microbiology and Leuven Food Science and Nutrition Research Centre (LFoRCe), KU Leuven, Leuven, Belgium; 2 Centre of Microbial and Plant Genetics, KU Leuven, Leuven, Belgium

**Keywords:** Applied microbiology, Bacteriology, Pathogens

## Abstract

*Clostridium botulinum* is an anaerobic sporeforming bacterium that is notorious for producing a potent neurotoxin. Spores of *C. botulinum* can survive mild food processing treatments and subsequently germinate, multiply, produce toxin and cause botulism. Control of spore germination and outgrowth is therefore essential for the safety of mildly processed foods. However, little is known about the process of spore germination in group II *C. botulinum* (gIICb), which are a major concern in chilled foods because they are psychrotrophic. The classical model of spore germination states that germination is triggered by the binding of a germinant molecule to a cognate germinant receptor. Remarkably, unlike many other sporeformers, gIICb has only one predicted canonical germinant receptor although it responds to multiple germinants. Therefore, we deleted the *gerBAC* locus that encodes this germinant receptor to determine its role in germination. Surprisingly, the deletion did not affect germination by any of the nutrient germinants, nor by the non-nutrient dodecylamine. We conclude that one or more other, so far unidentified, germinant receptors must be responsible for nutrient induced germination in gIICb. Furthermore, the *gerBAC* locus was strongly conserved with intact open reading frames in 159 gIICb genomes, suggesting that it has nevertheless an important function.

## Introduction

*Clostridium botulinum* is a heterogeneous species of sporeforming, strictly anaerobic bacteria that thrive in decaying organic matter in the soil or in aqueous environments. It is notorious for its ability to produce a potent neurotoxin that can cause botulism, a severe neuroparalytic disease^1–3^. Human botulism is predominantly associated with group I and II *C. botulinum* (gICb and gIICb), and these groups have therefore been studied most extensively^4–6^. gIICb strains, in particular, are a major concern for the safety of mildly heat-processed refrigerated foods because they are psychrotrophic. Food producers have to ensure that the formulation of the food in combination with the storage conditions prevent multiplication of gIICb during the shelf-life of these foods^3,7–10^. Since spore germination necessarily preceeds vegetative cell growth, understanding the spore germination process is essential to accurately predict the probability of toxin formation. In addition, it may pave the way towards novel control strategies aimed at inhibiting or retarding spore germination.

In the natural environment, spore germination is induced when specific nutrients bind to spore germinant receptors (GRs) in the spore membrane, thus signaling that conditions are favorable for outgrowth. This sets off a self-propagating and irreversible cascade of events starting with the release of monovalent cations H^+^, Na^+^, K^+^ and Ca^2+^-dipicolinic acid (Ca^2+^-DPA) from the spore core, followed by degradation of the spore cortex and core rehydration. The latter is a key turning point in the spore germination process because it causes the loss of many of the spores’ resistance properties and at the same time triggers the onset of metabolism^11–13^.

The nutrients that trigger germination are species and strain specific, but most commonly include one or more L-amino acids, purine ribosides and D-sugars^14^. GRs designated as Ger receptors have been first identified in *B. subtilis* and later shown to be conserved in almost all sporeforming Bacilli and Clostridia. However, the diversity among Ger receptors is large. In *B. cereus*, a recent study defined 11 different phylogenetic clusters of Ger receptors, with the number of receptors per strain varying from four to ten^15^. The Ger receptor content is believed to reflect the spectrum of germinants that a strain responds to. In *B. subtilis* strain 168, for example, GerA responds to D-ala, while GerB and GerK cooperatively respond to a germinant mixture of L-asparagine, D-glucose, fructose and K^+^. However, the relation between the germination response and the presence of specific Ger receptors is usually more complex, as exemplified by the study of Warda *et al*.^15^, who failed to establish Ger receptor specificities in 17 *B. cereus* strains with a variable Ger receptor content^15^. Knockout analysis has also indicated that Ger receptors of the same type may have a different contribution to the germinant response in different bacteria. For example, GerL was linked to L-alanine germination in *B. cereus* 569, but did not affect L-alanine germination in *B. cereus* ATCC 14579^16^. Individual knockout of all *ger* operons in *B. cereus* ATCC 14579 failed to identify a role in germination for three (GerK, GerL and GerS) out of the seven Ger receptors, although all operons were transcribed during sporulation^16^. Clarification of the structure-function relationship of Ger receptors has also been hindered by the difficulty to isolate functional receptors, because they are membrane complexes composed of three subunits and associated with other proteins in a so-called germinosome complex^17,18^.

The three protein subunits constituting Ger-type GRs are usually encoded in a tricistronic operon. The A protein comprises four to eight predicted membrane-spanning domains, as well as large N- and C-terminal hydrophilic domains. The B protein contains seven to twelve transmembrane helices, and is structurally related to a superfamily of membrane-associated single-component membrane transporters, although the sequence similarity is low. The C subunit of the Ger receptor is predicted to be a lipoprotein that is anchored to the outer surface of the spore inner membrane^11,13,19^. Remarkably, while the available evidence indicates that all subunits are required to form a functional receptor in bacilli, several clostridia, like *C. beijerincki*, *C. butyricum*, *C. asparagiforme*, encode only a single A subunit, but it is unclear whether this subunit is a functional GR in these bacteria^11^.

Brunt *et al*.^20^ recently made an inventory of the Ger gene clusters in groups I-IV of *C. botulinum*, based on *in silico* analysis of 148 *C. botulinum* and 8 *C. sporogenes* genomes^20^. The latter were included because of their close relatedness to gICb. Four different *ger* clusters were identified (*gerX1-4*), eventually further divided into subtypes designated with an additional letter. gICb and *C. sporogenes* strains typically contain three to five different Ger receptors, encoded by *gerX1a*/*c*/*d*, *gerX2a/b/c* and/or *gerX3a*. In general, spores from these strains germinate in response to various amino acids in combination with L-lactate, although the latter is not always essential^21,22^. The functionality of *C. botulinum* Ger receptors has been experimentally studied in gICb strain ATCC 3502, which has a *gerX1a, gerX1d* and *gerX2b* cluster, and in *C. sporogenes* ATCC 15579, which has a *gerX1a, gerX1d, gerX2c and gerX3a* cluster^22^. Construction and analysis of insertional knockouts in the A subunit encoding gene of each individual *ger* cluster revealed that both *gerX1a* and *gerX1d* were essential for amino acid germination in the gICb strain, while *gerX2b* was completely dispensable. In the *C. sporogenes* strain, in contrast, only *gerX1d* was essential for germination, while *gerX1a* was dispensable, and *gerX2c* and *gerX3a* influenced the rate, but not the extent of germination. No conclusions could be made concerning the germinant specificity of the receptors, since the effect of knocking out a receptor was always the same for each of the germinants used (L-ala, L-cys, L-meth, L-ser, L-phe; each in combination with L-lactate). In *C. sporogenes*, four triple knockout mutants, each carrying only one intact *ger* cluster, were also constructed. Only the mutant with an intact *gerX1d* cluster retained wild-type germinant responsiveness, while all other mutants failed to germinate. While this study yielded important insights in Ger receptor function, it also has an important limitation because the insertional knockout of the A subunit gene in a *ger* cluster does not necessarily abolish expression of the B and C subunits. This is certainly the case for the *gerX3a* cluster, which has a bicistronic organization, with *gerB* transcribed in the opposite direction as *gerA* and *gerC*.

Much like gICb, gIICb spores germinate in response to several amino acids in combination with L-lactate, but other than in gICb, L-lactate seems to be essential for germination. In a systematic study with three gIICb strains, L-alanine, L-cysteine and L-serine were the amino acids that induced the strongest germination response^23^. In addition, it was already reported earlier that gIICb spores germinated in response to amino acids at pH 9 in absence of lactate, as well as the combination of L-alanine with glucose, galactose or maltose at neutral pH^24^. Analysis of gIICb genome sequences indicates that they encode only one Ger receptor, corresponding to the *gerX3b* type^20^, but the function of this receptor remains to be investigated.

Mutational studies have also been conducted in *C. perfringens*, which contains a *gerX3* like locus designated *gerK*, and a distantly located monocistronic *gerAA* gene^25,26^. It was concluded that GerKA, GerAA and GerKB only play auxiliary roles since inactivation of either of the corresponding genes had no significant effect on germination. GerKC, on the other hand, was required for the response to germinants such as KCl, L-asparagine, and a L-asparagine–KCl mixture. The finding that the A and B subunits are dispensable in *C. perfringens* is in striking contrast with the situation in *B. subtilis*^27^.

While Ger-type receptors are widely distributed in sporulating bacteria, there is evidence for the existence of other receptor types. The genome sequences of at least two *Clostridium* species, *C. bartletti and C. difficile*, do not contain Ger gene homologs, although spore germination in these bacteria is also induced by specific germinants, consistent with a mechanism involving specific receptors. In the case of *C. difficile*, germination is induced by the bile component taurocholate, which signals to the spores that they are in their primary niche, the animal gut. Based on the analysis of germination mutants, Francis *et al*. (2013) proposed that a catalytically inactive homolog of the germination protease CspC is the GR^28^.

Besides nutrients, also some non-nutrients can induce spore germination. Exogenous Ca^2+^-DPA directly activates the cortex hydrolase CwlJ in *Bacillus* without the involvement of GRs^29^. However, Ca^2+^-DPA germination is not always clearly linked to CwlJ, since *C. difficile* spores do not germinate with Ca^2+^-DPA although the organism encodes a CwlJ homolog (30% amino acid identity with *B. subtilis* CwlJ)^30^, while *C. perfringens* spores germinate in response to Ca^2+^-DPA despite the absence of a *cwlJ* homolog in the genome. In *C. perfringens*, GerK appears to mediate the Ca^2+^-DPA response since GerK deficient spores germinated very poorly with Ca^2+^-DPA^26^. Finally, the cationic surfactant dodecylamine has also been described as a non-nutrient inducer of germination in *B. subtilis*, *C. perfringens* and *C. difficile*^31–33^. Dodecylamine was proposed to open the spore’s Ca^2+^-DPA channels without the involvement of the GRs or cortex hydrolases.

It is clear from the above that the mechanisms of spore germination can vary substantially between bacteria and depend on the germinant, and that in clostridia in particular, the role of the Ger receptors and the possible existence of additional GRs requires further investigation. In gIICb strains, the presence of only a single Ger-type receptor (GerX3b) contrasts with the large variety of amino acids that can trigger germination. It seems unlikely that this receptor can respond specifically to all these germinants. In the present work, we deleted the entire *gerBAC* locus encoding GerX3b to analyse its role in germination by different nutrient and non-nutrient germinants. This was done in a mutant of gIICb strain NCTC 11219 from which we previously deleted the *bont/E* toxin gene^34^. Gene deletion is still a major technical challenge in gIICb, and the present work is only the second (after the deletion of *bont/*E) in which this is successfully accomplished.

## Material and Methods

### Bacterial strains and growth conditions

This work was conducted in an atoxigenic (Δ*bont*::*ermB*) and uracil auxotrophic (Δ*pyrE*) mutant of group II *C. botulinum* strain NCTC 11219 type E, further in this manuscript shortly named Δ*bont* Δ*pyr* or parent strain^34^. This strain was used for biosafety reasons, and because of the possibility to use *pyrE in trans* as a negative selection marker for replacement of the *gerBAC* locus. Liquid clostridial cultures were grown at 30 °C in trypticase peptone glucose yeast extract broth (TPGY), composed of 50 g/l trypticase (Becton-Dickinson, MD, USA), 5 g/l bacteriological peptone (Oxoid, Basingstoke, UK), 20 g/l yeast extract (Oxoid), 4 g/l glucose (Acros, New Jersey, USA) and 1 g/l sodium thioglycollate (Sigma, Steinheim, Germany). Solid culture media were reinforced clostridial medium (RCM) agar, composed of 37 g/l RCM (Thermo Scientific, Darmstadt, Germany) and 15 g/l agar, TPGY agar (TPGY broth + 15 g/l agar), or tryptone yeast extract thioglycollate (TYG) agar, composed of 30 g/l tryptone (Lab M, Heywood, UK), 20 g/l yeast extract, 1 g/l sodium thioglycollate and 15 g/l agar. Vegetative cultures were always grown and manipulated under strictly anoxic conditions in a Don Whitley DG250 anaerobic workstation operating with a gas mixture of 80% N_2_, 10% CO_2_, and 10% H_2_, and using overnight pre-reduced media. Spore suspensions were handled in open air, and transferred to the workstation only for experiments involving outgrowth. Spore suspensions were prepared using a two-phase sporulation medium and stored in 0.85% NaCl at 4 °C as described previously^34^. *E. coli* strains were grown in lysogeny broth (LB; 10 g/l tryptone, 5 g/l yeast extract, 5 g/l NaCl) or on LB agar (LB + 15 g/l agar) at 37 °C. *E. coli* DH5α was used for cloning and maintenance of plasmids, while *E. coli* CA434 (HB101 containing plasmid R702^35^) served as donor for plasmid conjugation. Antibiotics (Applichem, Darmstadt, Germany) were added at the following concentrations when appropriate: thiamphenicol (Tm, 15 μg/ml in agar, 7.5 μg/ml in broth), spectinomycin (Sp, 600 μg/ml for *C. botulinum*, 100 µg/ml for *E. coli*), cycloserine (Cy, 250 μg/ml), chloramphenicol (Cm, 25 μg/ml in agar, 12.5 μg/ml in broth). 5-Fluoroorotic acid (5-FOA, Manchester Organics, Cheshire, UK) was used at 500 µg/ml to screen for loss of the *pyrE-*expressing plasmid pMTL84151∆*gerBAC*.

### Plasmid construction

The plasmid pMTL84151∆*gerBAC* was constructed to replace the *gerBAC* locus with the Sp resistance marker *aad9* by double homologous recombination in *C. botulinum* NCTC 11219 Δ*bont* Δ*pyr*. All primers used are listed in Table 1 and were obtained from Integrated DNA Technologies (Heverlee, Belgium). First, the entire *pyrE* open reading frame (675 bp) was amplified with primers pyrE11219_F and pyrE11219_R, restricted with NdeI/SacI and placed after the p_fdx_ promotor in pMTL83353 which had been opened with the same enzymes. Hereafter, the fragment containing p_fdx_ and *pyrE* was amplified with primers pMTL83353_F and pyrE11219_R, digested with SbfI and SacI and cloned in pMTL84151, opened with the same enzymes. Flanking regions of *gerBAC* (5′ region: 932 bp, 3′ region: 1091 bp) were amplified from NCTC 11219 DNA using primer pairs ger5′F/ger5′R and ger3′F/ger3′R, respectively. The *aad9* locus (1009 bp) was amplified from plasmid pMTL83353 with primers aad9_F and aad9_R. The flanking 5′ and 3′ regions, the *aad9* fragment and pMTL84151 containing p_fdx_-*pyrE*, opened by PCR using primer pair pMTL84151_openF / pMTL84151_openR, were then cloned together using Gibson assembly, following the suppliers protocol (New England Biolabs, Hitchin, UK). After verification by PCR and sequence analysis, the resulting plasmid construct was designated pMTL84151∆*gerBAC* and was transferred into *E. coli* CA434 by electroporation.

**Table 1:** Oligonucleotides used for cloning and construct verification.

Name	Sequence (5′-3′)
pyrE11219_F	AGGCATATGGAAGCATATAAAAAAGAG
pyrE11219_R	CTTGAGCTCCTACTTAGCACCATATTC
pMTL83353_F	GAGCCTGCAGGATAAAAAAATTGTAG
pMTL84151_openR	ttctggtgatttaactttagCTCCTACTTAGCACCATATTC
pMTL84151_openF	ccgtcgttttacaacgtc
ger5′F	gatgaaattaaaactagaatagatgaatattacaaagaatatggtgctaagtaggagCTAAAGTTAAATCACCAGAAGG
ger5′R	GCACTTTACTTATACATATATCACTAATGAC
ger3′F	ATGAAGGTATAATTTTAAAGATGCTCTAAAATCTC
ger3′R	acgacgttgtaaaacgacggCTAAACATTTCTCTACATCTGC
aad9_F	tctttattttagtcattagtgatatatgtataagtaaagtgcCAATGAATAGGTTTACACTTACTTTAGTT
aad9_R	aataacagagattttagagcatctttaaaattataccttcatAATAAAACAAAAAAATTGAAAAAAGTGTTTCCACCA
gerA_F	GTTACATAGAAGGAGTGGCACC
gerA_R	AAATGCAAGCCATCTTAACACTCTC
∆gerBAC_upF	GTTATAGCATGTAAATCAACCACGC
∆gerBAC_downR	TCTTAGCTCCATTAATTTCAGCAC

Restriction sites are underlined: NdeI (CATATG), SacI (GAGCTC) and SbfI (CCTGCAGG). The small letters indicate the overhang region of the primer, necessary for annealing fragments via Gibson assembly.

### Construction of the ∆*gerBAC* deletion mutant

Plasmid pMTL84151∆*gerBAC* was introduced into NCTC 11219 Δ*bont* Δ*pyr* by conjugation as described previously^34^, using selection on RCM agar with Tm and Cy. Purified transconjugants were resistant to Sp and sensitive to FOA, confirming expression of the plasmid *aad9* resistance marker and *pyrE* gene, respectively.

Transconjugants grown in TPGY with Sp, were then plated on TPGY with Sp and FOA to select for clones in which double homologous recombination with the flanking loci of *gerBAC* as well as loss of the plasmid had occurred. Several cultures had to be grown and plated in parallel before a culture was found that yielded Sp and FOA resistant colonies. These colonies had also lost Tm resistance, indicating loss of the plasmid. PCR and sequence analysis with primers ∆gerBAC_upF/∆gerBAC_downR, annealing just outside the flanking regions used for homologous recombination, confirmed that *gerBAC* was replaced by *aad9*. Another PCR with internal primers of *gerA* was used to verify that the *gerBAC* locus had not translocated elsewhere in the genome. The mutant was designated NCTC 11219 Δ*bont* Δ*pyr* Δ*gerBAC*::*aad9* (in this manuscript further described as Δ*bont* Δ*pyr* Δ*gerBAC*).

### Whole genome sequence assembly and analysis

To exclude the very unlikely possibility that the *gerBAC* genes had moved to another place in the chromosome, the Δ*bont* Δ*pyr* Δ*gerBAC* strain and its parent Δ*bont* Δ*pyr* were subjected to whole genome sequence analysis on a Illumina MiSeq sequencer. First, gDNA from both strains was isolated from overnight cultures using the GeneJET Genomic DNA purification kit (Thermo Scientific). DNA purity and concentration was assessed by Nanodrop analysis, gel electrophoresis and Qubit (Thermo Scientific) analysis. Paired-end libraries were constructed using the NEBNext Ultra gDNA library prep protocol with an average insert size of 240 bp, and analyzed on the Agilent BioAnalyzer (VIB nucleomics core, Belgium) resulting in on average 1.2 million reads per sample. Reads were analysed with Qiagen’s CLC Genomics Workbench version 8.5 (http://www.clcbio.com/), and subjected to standard quality control, read trimming and filtering (reads <15 nucleotides were discarded, quality score limit = 0.01, ambiguous nucleotides trim limit = 2), and read mapping to *C. botulinum* NCTC 11219 reference WGS with accession number JXMR00000000 (using parameters: mismatch cost = 2, insertion cost = 3, deletion cost = 3, length fraction = 0.8, similarity fraction = 0.8). This resulted in an average 43-fold genome coverage for the parental strain and 51-fold coverage for the Δ*gerBAC* mutant.

### Chemicals and stock solutions

Nutrient germinant mixtures were freshly prepared as 2x concentrated solution in 100 mM Tris-HCl buffer pH 7.4, from the following chemicals: L-alanine (Sigma), L-lactate sodium salt (Acros), NaHCO_3_ (Acros), L-serine (Acros), L-cysteine (Acros), L-threonine (Acros), inosine (Sigma) and D-glucose (Acros). Dodecylamine (Acros) was first dissolved at 1 M in ethanol, and then diluted to 2x of the concentration used in germination experiments (6 mM) in 100 mM Tris-HCl buffer. Stock solutions of 120 mM CaCl_2_ (Chem Lab, Belgium) and 120 mM DPA (Acros) were made in 100 mM Tris-HCl (pH 7.4), and the pH of the DPA solution was readjusted to pH 7.5 with Trizma base (Sigma-Aldrich). The two solutions were then mixed in a 1:1 volume ratio and eventually further diluted in 100 mM Tris-HCl (pH 7.4) to achieve final Ca^2+^-DPA concentrations of 60 mM, 50 mM, 30 mM and 20 mM.

### Germination assays

Immediately before use, spores were collected by centrifugation (6,000 RPM, 10 min, 4 °C), resuspended in 100 mM Tris-HCl buffer (pH 7.4), and heated for 10 min at 65 °C to inactivate any residual vegetative cells and spores that would have spontaneously germinated during storage, and also to activate dormant spores for germination. Hereafter a sample was taken from the suspension, diluted in Tris-HCl buffer and plated on TPGY to determine the initial spore count (t0-HT), which was always around 7 log cfu/ml. The remainder of the spore suspension was mixed with an equal volume of a 2x germinant solution (or buffer as a negative control), and incubated for 4 h at 30 °C. The suspension was then heated for 10 min at 65 °C and plated on TPGY (t4-HT). The degree of germination was expressed as log(t0-HT) – log (t4-HT). Ca^2+^-DPA was used at 20–60 mM for germination experiments, but because 2x concentrated solutions of 120 mM cannot be made due to the limited solubility of Ca^2+^-DPA, the spores were resuspended immediately in 60 mM, 50 mM, 30 mM and 20 mM Ca^2+^-DPA, and the initial heat treatment to activate the spores was done in the presence of Ca^2+^-DPA in this case.

### DPA measurement

After 4 h of incubation, spores were removed from the germinant solution by centrifugation (12,000 × g for 5 min), and 100 µl of the supernatant was mixed with an equal volume of 20 mM TbCl_3_ in a 96-well black microtiter plate (Greiner Bio-one, Belgium)^36^. Fluorescence measurements were done in a spectrofluorometer (Synergy Mx-biotek, USA) with excitation and emission wavelengths of 270 and 545 nm, respectively. DPA release was expressed relative to the total spore DPA, which was measured after treatment of the spores at 98 °C for 30 min^33^.

### Microscopy

Heat activated spores were immobilized on thin pads of 1% agarose in Tris-HCl buffer on glass slides, with or without the germinants L-alanine/L-lactate/NaHCO_3_ (all at 50 mM) incorporated in the agar pad. Time-lapse microscopy was performed with a Ti-Eclipse inverted microscope (Nikon, France) equipped with a pE-100 camera (CoolLED, Andover, UK). Images were acquired using NIS-Elements (Nikon) and further handled with the open source software ImageJ.

### Statistical analysis

Experiments were conducted in three repetitions with independent spore suspensions unless otherwise mentioned, and statistical analysis of germination and DPA release was performed using the two-tailed Student’s T-test with a significance level of 0.05.

### *In silico* analysis of Ger receptor genes in 152 gIICb strains

The raw Illumina reads of a previously published^37^ diverse set of 152 gIICb strains were retrieved from the NCBI Sequence Read Archive database (Accession number: SRP059342, no corresponding assembly available). The fastq files were processed with BBduk for removal of adapter contamination, trimming (Phred score >28), and size exclusion (read length >50 bp). Each fastq file was subsequently inspected with FastQC for quality control^38^. The genome of each strain was assembled with SPAdes^39^, and the quality of the assembly assessed with QUAST^40^. Functional annotation was done using Prokka^41^ with a custom protein database created from strains of the *Clostridium* genus. The protein content of each genome was finally queried using blastp against the GerX3b subunits present in strain Eklund 17B (gene locus tags for *gerC*, *gerA*, *gerB*: CLL_A3167, CLL_A3168, CLL_A3169).

## Results

### Construction of a ∆*gerBAC* deletion mutant

**Figure 1:**
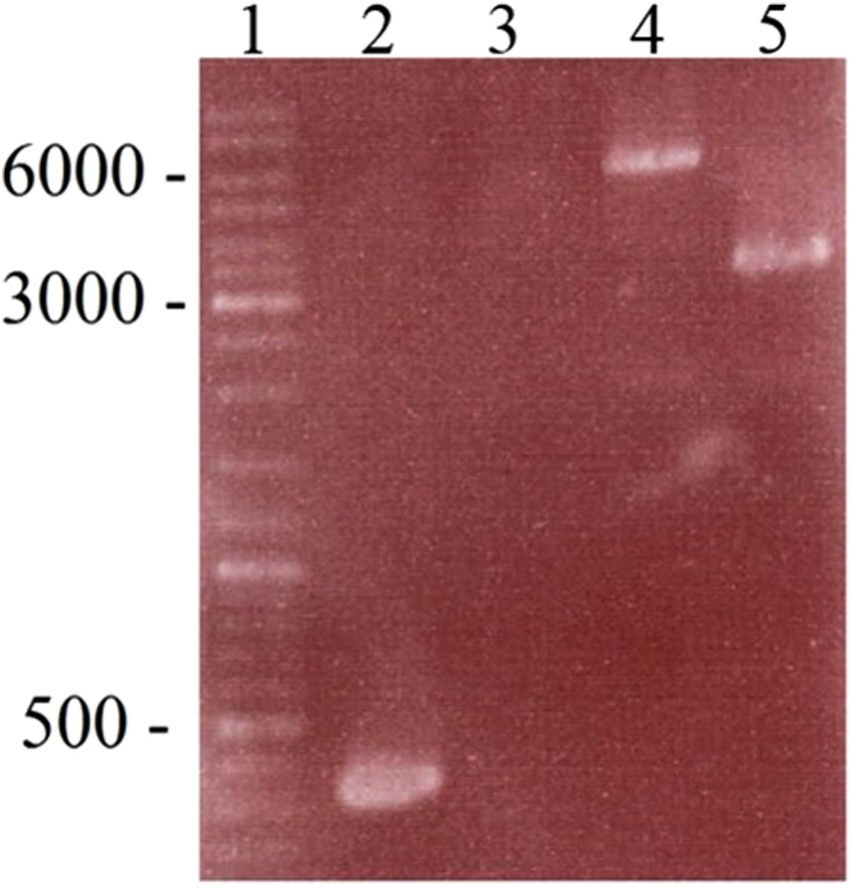
PCR analysis on the *gerBAC* region of the parental strain NCTC 11219 Δ*bont* Δ*pyr* and the Δ*bon*t Δ*pyr* ∆*gerBAC* mutant. Lane 1: Molecular size marker (GeneRuler from Thermo Scientific); Lane 2: Parental strain, internal fragment of gerA amplified with primers gerA_F and gerA_R (expected size: 329 bp). Lane 3: ∆*gerBAC*, no internal *gerA* fragment could be amplified with primers gerA_F and gerA_R. Lane 4: Parental strain, *gerBAC* locus amplified with primers ∆gerBAC_upF and ∆gerBAC_downR (expected size: 6252 bp). Lane 5: ∆*gerBAC, gerBAC* locus amplified with primers ∆gerBAC_upF and ∆gerBAC_downR (expected size: 3198 bp).

The deletion of the *gerBAC* locus was done in strain NCTC 11219 Δ*bont* Δ*pyr* which was constructed previously in our group^34^. Besides having the advantage of being nontoxigenic, the *pyrE* deletion renders this strain resistant to FOA, making it possible to use the *pyrE* gene as a negative selection marker for making additional gene replacements. The *pyrE* deletion also renders the strain auxotrophic for uracil, but this did not affect its growth in TPGY medium. Plasmid pMTL84151∆*gerBAC* (Tm^R^Sp^R^) was conjugated to Δ*bont* Δ*pyr* to allow replacement of *gerBAC* with *aad9*. Since *pyrE* is expressed on the plasmid, it renders the strain FOA-sensitive. Then, plating on RCM with FOA and Sp selects for clones that had lost the plasmid but at the same time retained the Sp resistance cassette by double homologous recombination. PCR analysis (Fig. 1) and sequencing of such clones confirmed replacement of *gerBAC* by *aad9*, and WGS analysis additionally confirmed absence of the entire *gerBAC* locus in the genome of strain NCTC 11219 Δ*bont* Δ*pyr* ∆*gerBAC*.

The spore yield of the ∆*gerBAC* strain (6.9 +/− 0.6 log cfu/ml; n = 6) was unaffected (p > 0.05) compared to that of the parental Δ*bont* Δ*pyr* strain (7.2 +/− 0.4 log cfu/ml; n = 6). Colony formation from spores of the ∆*gerBAC* strain on TPGY agar was not delayed and the colonies were indistinguishable from those of the parent strain. Spore suspensions had stable spore counts as determined after a heat treatment for at least 4 months at 3 °C.

### The role of GerX3b in germination induced by L-alanine/L-lactate/NaHCO_3_

**Figure 2:**
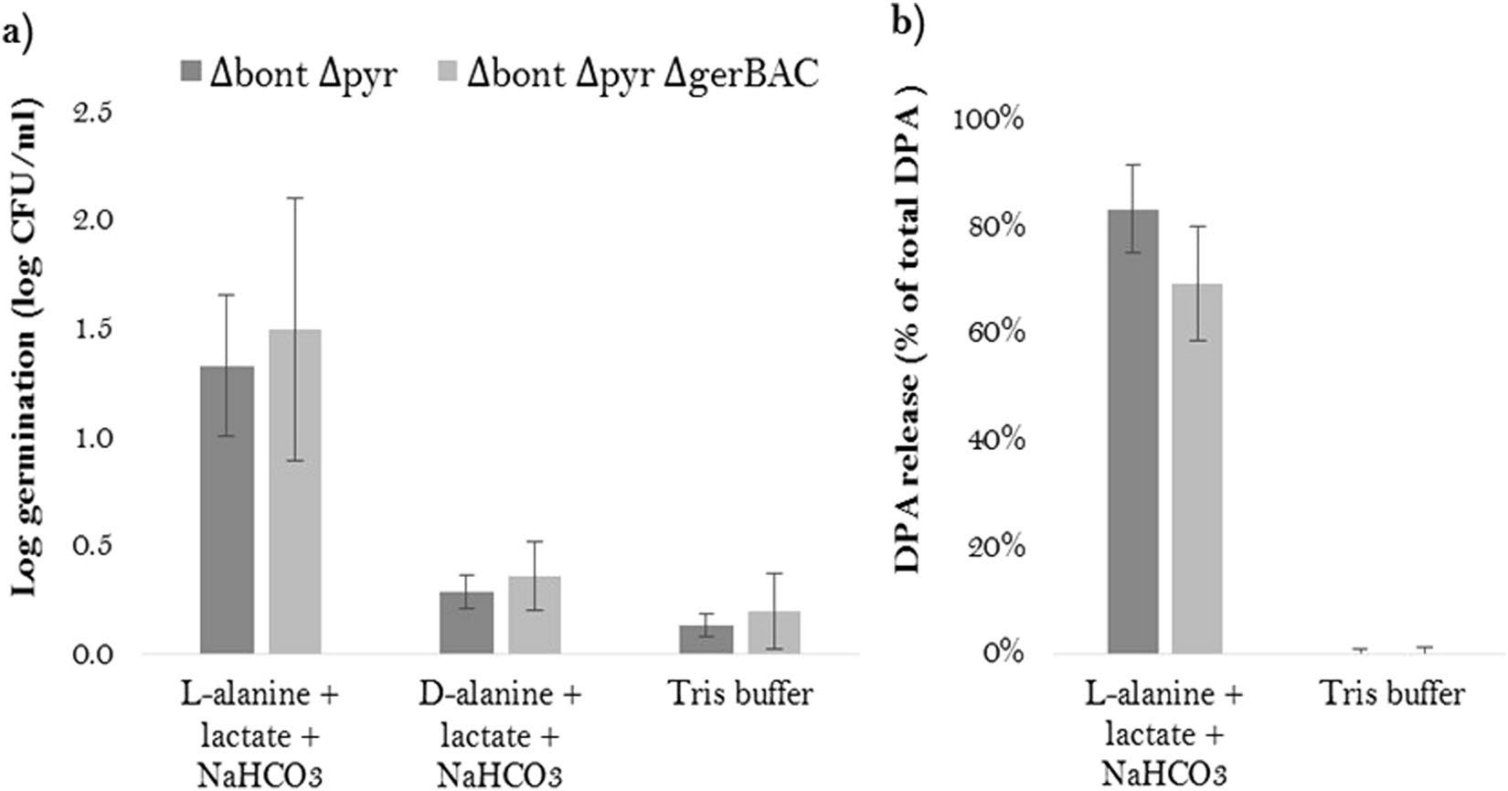
Spore germination of ∆*gerBAC* spores compared to the parental strain, in L-ala/L-lact/NaHCO_3_ (all at 50 mM) in 100 mM Tris-HCl buffer (pH 7.4). (**a**) Germination assessed by loss of heat resistance (65 °C/10 min), after incubation of heat-activated spores for 4 h at 30 °C in the germinant mixture. D-alanine (50 mM) was used to demonstrate the stereospecific action of germination induction in comparison to L-alanine. Means +/− standard deviations are shown of three experiments using independent spore crops. No significant differences were found (p > 0.05) between the two strains. (**b**) DPA release of heat-activated spores incubated for 4 h at 30 °C in the germinant mixture, relative to the total DPA content. Mean percentages +/− standard deviations are shown of three experiments using independent spore crops. No significant differences were found (p > 0.05) between the two strains.

**Figure 3:**
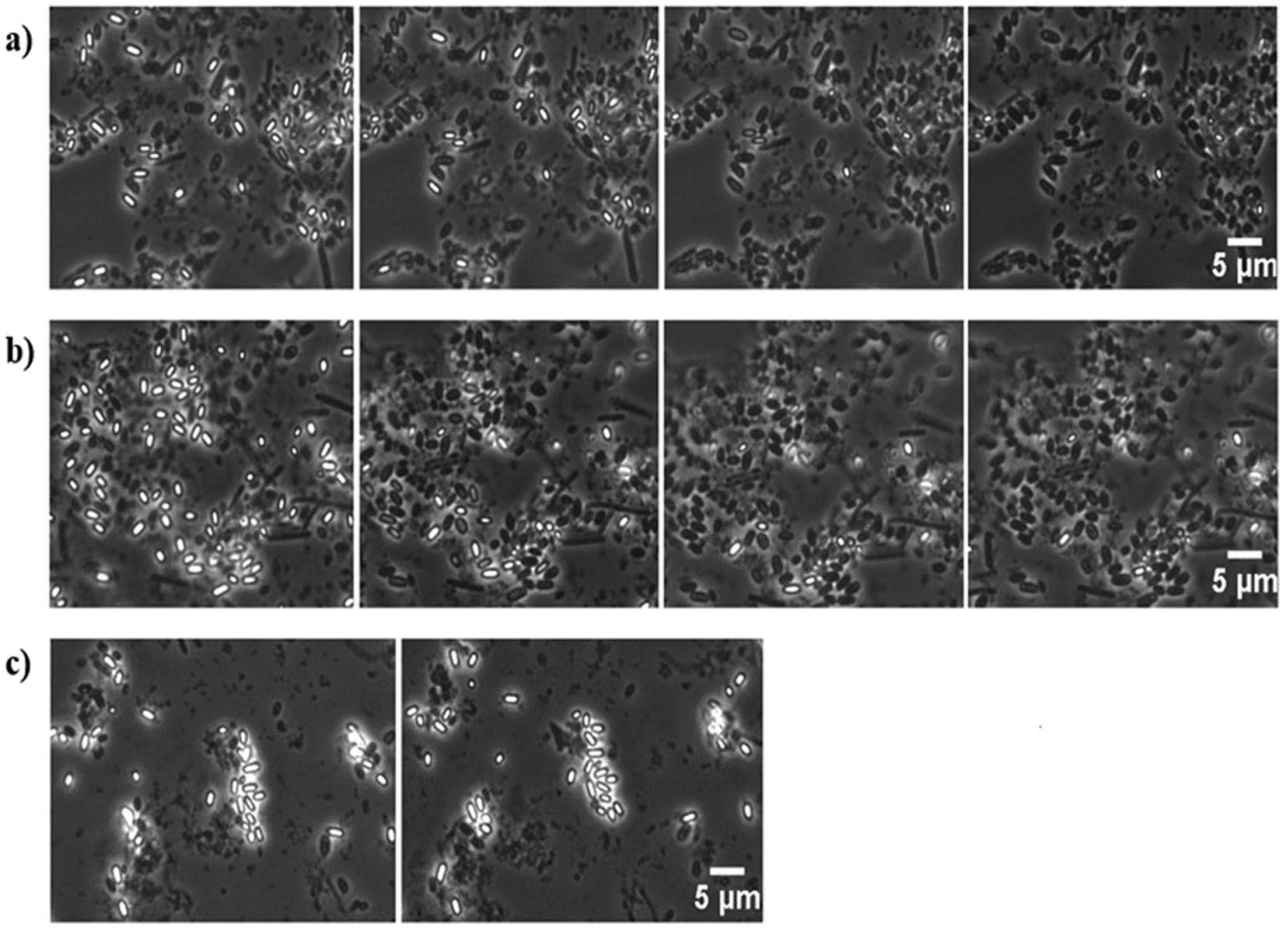
Spore germination of ∆*gerBAC* spores and the parental strain visualized with time-lapse phase-contrast microscopy. (**a**) Spores of the parental strain and (**b**) the ∆*gerBAC* mutant 5 min, 20 min, 40 min and 1 h (from left to right) after deposition on the agar pad containing the germinants L-ala/L-lact/NaHCO_3_ (all at 50 mM). (**c**) Spores of the ∆*gerBAC* mutant on an agar pad without germinants 5 min (left) and 1 h (right) after deposition on the pad. Spores of parental strain also remained bright (data not shown).

Since the combination of L-alanine, L-lactate and NaHCO_3_ is one of the most commonly used and efficient inducers of spore germination in gIICb^23,24^, we first used this germinant mixture to analyse the impact of deletion of the GerX3b receptor. Unexpectedly, germination of the mutant, as assessed by heat treatment and plating and by DPA release (1.5 +/− 0.6 log germination; 69.3 +/− 10.7% DPA release), was not significantly different from that of the parent strain (1.3 +/− 0.3 log germination; 83.2 +/− 8.2% DPA release) (Fig. 2). Replacement of L-alanine by D-alanine reduced germination of both strains to the background level observed in the absence of germinants, indicating stereospecificity of the L-alanine response. Germination was not initiated by L-alanine and NaHCO_3_ in the absence of L-lactate (data not shown), in accordance with previous reports^20,23^. Germination was also monitored by time-lapse phase-contrast microscopy of spores deposited on agar pads with and without the L-alanine/L-lactate/NaHCO_3_ germinant mixture. The results indicated that spore germination progressed at a similar rate in the parental and Δ*gerBAC* spores (Fig. 3a and b). By the time the spores could be viewed (5 min after deposition on the pad), there were already some phase-dark spores visible. The phase-dark fraction further increased after 20 and 40 min, and after 60 min only few refractile spores remained. When no germinants were included in the pads, the spores remained fully bright (Fig. 3c).

### The role of GerX3b in germination induced by other nutrient germinants

Because deletion of the putative GerX3b receptor did not affect germination by L-alanine/L-lactate/NaHCO_3_, we tested other nutrient mixtures previously described to induce gIICb spore germination (Table 2). Only mixtures that induced at least a 0.5 log germination of the parental strain were subsequently also tested on the Δ*gerBAC* mutant. Besides L-alanine, both L-serine and L-cysteine in combination with L-lactate and NaHCO_3_ were previously reported as very efficient inducers of germination of gIICb spores^23,24^, and this was confirmed for the spores of our strain. L-threonine/L-lactate/NaHCO_3_ and inosine/L-alanine/NaHCO_3_ mixtures, previously reported as moderate germination inducers^23,24^, were about equally effective.

**Table 2:** Spore germination of NCTC 11219 strains after 4 h at 30 °C in different germinant mixtures.

Nutrient mixture in 100 mM Tris-HCl buffer pH 7.4	Log germination (log CFU/ml)		Reference
	∆*bont* ∆*pyr*	∆*bont* ∆*pyr* ∆*gerBAC*	
L-alanine + L-lactate + NaHCO_3_ (all 50 mM)	1.3 +/− 0.3	1.5 +/− 0.6	23
L-serine + L-lactate + NaHCO_3_ (all 50 mM)	2.0 +/− 0.0	2.0 +/− 0.2	23
L-cysteine + L-lactate + NaHCO_3_ (all 50 mM)	1.0 +/− 0.1	1.4 +/− 0.5	23
L-valine + L-lactate + NaHCO_3_ (all 50 mM)	1.1 +/− 0.6	1.2 +/− 0.3	
L-isoleucine + L-lactate + NaHCO_3_ (all 50 mM)	0.7 +/− 0.3	0.8 +/− 0.1	
L-threonine (100 mM) + L-lactate (50 mM) + NaHCO_3_ (50 mM)	1.3 +/− 0.6	1.0 +/− 0.5	23
Inosine (20 mM) + L-alanine (25 mM) + NaHCO_3_ (60 mM)	1.3 +/− 0.4	1.3 +/− 0.4	23,24
Glucose (20 mM) + L-alanine (25 mM) + NaHCO_3_ (60 mM)	0.1 +/− 0.2	ND	24
Glycine (50 mM)	0.1 +/− 0.2	ND	24
Glycine + L-lactate + NaHCO_3_ (all 50 mM)	0.1 +/− 0.3	ND	23
**Nutrient mixture in 100 mM carbonate buffer pH 9.0**	**Phase-dark spores**		
	**∆*bont*** **∆*pyr***	**∆*bont*** **∆*pyr*** **∆*gerBAC***	
L-alanine (100 mM) + NaHCO_3_ (50 mM)	<5%	ND	24
L-cysteine (100 mM) + NaHCO_3_ (50 mM)	<5%	ND	24
L-serine (100 mM) + NaHCO_3_ (50 mM)	<5%	ND	24
L-threonine (100 mM) + NaHCO_3_ (50 mM)	<5%	ND	24
**Non-nutrient mixture in 100 mM Tris-HCl buffer pH 7.4**	**Log germination (log CFU/ml)**		
	**∆*bont*** **∆*pyr***	**∆*bont*** **∆*pyr*** **∆*gerBAC***	
Ca^2+^-DPA (60 mM)^a^	0.2 +/− 0.3	ND	25,29
Dodecylamine (3 mM)	3.3 +/− 0.6	3.9 +/− 1.1	33,42

The degree of germination was determined by plate counting heated spore suspensions (65 °C/10 min), except for the treatments in carbonate buffer at pH 9.0, for which germination was evaluated by phase-contrast microscopy. Data for L-alanine/L-lactate/NaHCO_3_ are from Fig. 1. (ND: not determined). Last column refers to studies in which mixtures were identified as gIICb germinant. ^a^Ca^2+^-DPA at 20, 30 and 50 mM, germination levels were 0.3 +/− 0.2, 0.1 +/− 0.3 and 0.2 +/− 0.1 log, respectively.

We additionally tested combinations of all other amino acids with L-lactate and NaHCO_3_, and found that only L-isoleucine and L-valine also induced >0.5 log germination. However, there was again no significant difference between the parental strain and the Δ*gerBAC* mutant with these germinants (p > 0.05).

No germination was observed with glucose/L-alanine/NaHCO_3_, glycine, and glycine/L-lactate/NaHCO_3_, although these were previously reported to induce germination (Table 2)^25^. Finally, Ando (1971) reported gIICb spore germination by L-alanine, L-cysteine, L-serine at pH 9.0 in the presence of NaHCO_3_ but without L-lactate^24^. However, we could not assess spore germination based on the loss of heat resistance for these mixtures, because the spores became heat sensitive in carbonate buffer (pH 9.0) even in the absence of nutrients. Therefore, germination was evaluated by microscopically evaluating the loss of refractility. Because the fraction of phase-dark spores remained <5% even after overnight incubation in the germinant mix, we concluded that these amino acids at pH 9.0 did not induce germination in the NCTC 11219 strain.

### The role of GerX3b in germination induced by non-nutrients

Because loss of the putative GerBAC receptor did not affect spore germination by any of the tested nutrient germinants, we next evaluated the effect on spore germination by the non-nutrient germinants Ca^2+^-DPA and dodecylamine. The ability of these compounds to induce spore germination had not yet been reported in gIICb. Ca^2+^-DPA failed to induce spore germination in the parental strain at all tested concentrations (20–60 mM), and was therefore not further tested on the ∆*gerBAC* mutant.

In contrast, spores incubated with dodecylamine (3 mM, 4 h at 30 °C) showed strongly reduced counts after heat treatment, suggesting induction of germination. However, there was again no significant difference (P > 0.05) between the parental strain and the ∆*gerBAC* mutant (Table 2). Furthermore, the dodecylamine-treated spores did not become fully phase-dark, and released only a relatively small amount of their DPA (42.4 +/− 3.2% for the ∆*gerBAC* mutant and 39.1 +/− 9.6% for the parental strain). Similarly low values of DPA release have been reported previously when dodecylamine is used to induce spore germination at relatively low temperature (30 °C)^34^. Together, these observations suggest that dodecylamine treatment may not induce a genuine physiological spore germination process, or that the germination process is incomplete.

### *In silico* analysis of Ger receptor genes in 152 gIICb strains

We assembled previously published sequence reads of 152 gIICb strains^40^. Seventeen strains were removed from the analysis due to poor assembly performance (N50 < 10,000) (Table S1). Functional analysis of the 135 remaining strains revealed that 130 strains carry the *gerBAC* locus, with apparently intact open reading frames of the three genes. In each of the five remaining strains, there was always one gene missing, but this was probably an artefact of the assembly because the neighbouring gerBAC gene(s) were at the edge of a contig in those cases. The percentages of positive-scoring substitutions, calculated by NCBI Blastp, varied between 96.30 and 100% (full data shown in Table S2).

## Discussion

The classical model of bacterial spore germination states that germination is triggered by the specific binding of a germinant molecule to a cognate GR in the spore membrane. The spores of most sporeforming bacteria respond to different nutrient germinants by means of an array of different GRs. The best studied GRs are those of the Ger-type. Since they are found in almost all sporeforming Bacilli and Clostridia and their importance for germination has been documented in several species, they are therefore considered to be the predominant, if not the only, GRs in these bacteria. This work is the first experimental study of GRs in group II *C. botulinum*. Analysis of whole genome sequences of 24 gIICb strains (18 of toxin type E, 4 of toxin type B, and 2 of toxin type F) previously indicated the presence of a single *ger* locus of the GerX3b type (*gerBAC*) in all these strains^20^. However, it is difficult to understand how this single receptor could have specific binding sites for the large variety of nutrients that can trigger spore germination in this organism. As a first step to unravel the precise role of the GerX3b receptor in germination, we therefore undertook to delete the entire *gerBAC* locus encoding the three receptor subunits, using a gene replacement technique that we applied previously to delete the *bont* gene^34^.

The entire *gerBAC* locus was successfully replaced by a spectinomycin resistance marker, as confirmed by sequencing of specific PCR amplicons of the region and whole genome sequence analysis. Much to our surprise, the deletion did not affect germination induction by any of seven major nutrient germinant mixtures, nor by the non-nutrient dodecylamine. Specifically for L-alanine (in combination with L-lactate), we demonstrated that the germination response is stereospecific, since D-alanine did not induce germination. This is in line with the notion that germinants, in gIICb as in other sporeformers, induce spore germination by interaction with a specific receptor. However, our results strongly suggest that GerX3b is not a functional GR, and thus lead to the conclusion that one or more other, so far unidentified, GRs must be responsible for nutrient-induced germination in gIICb.

One possible alternative receptor are the CspC orthologues, since a non-catalytically active CspC variant has been proposed to act as GR in *C. difficile*^28^. *cspC* and *cspBA* are located directly upstream of *sleC* in *C. difficile*, and the gene upstream of *sleC* in *C. botulinum* NCTC 11219 also encodes a predicted subtilase family protein, although the similarity to CspC from *C. difficile* is rather low (32% amino acid identity over 76% of the sequence). In addition, this putative protease is predicted to contain an intact Asp/Ser/His catalytic triad, as opposed to CspC of *C. difficile* in which two of the three catalytic residues are absent. BLAST analysis revealed five additional gene products in *C. botulinum* NCTC 11219 showing low but significant similarity to CspC of *C. difficile* R20291. In the genomes of gIICb strains Beluga, Alaska E43 and Eklund 17B, the number of CspC orthologues is 5, 6 and 10, respectively. All these predicted proteins are annotated as members of the subtilase family, and contain an intact catalytic triad (data not shown).

Since our results suggest that GerX3b is not a functional GR, and in the assumption that this is the case in all gIICb strains, one would expect the *gerBAC* genes to have accumulated loss of function mutations in some strains. Brunt *et al*.^20^ already analyzed the genome sequences of 24 gIICb strains and found that they all had intact *gerBAC* genes^20^. We extended this analysis with 135 additional strains and similarly found that all had intact *gerBAC* genes. Thus, it appears that maintenance of an intact *gerBAC* locus is important in gIICb. We are not aware that alternative functions have been reported for Ger-type receptors in any sporeforming bacteria, and it will therefore be interesting to explore such functions in gIICb. On the other hand, the quest for the genuine GRs in gIICb is open. As discussed above, the CspC-like proteins are possible candidates, and their large number in gIICb is compatible with the large variety of germinants in gIICb. However, if they show functional redundancy and hierarchy, as is the case for the multiple Ger receptors in *B. cereus*, their functional analysis will be a difficult task, because making (multiple) gene knockouts in gIICb remains a challenge. On the other hand, the possible existence of an entirely novel class of GRs should also be considered, and it would be worthwhile to isolate and analyse germination mutants to investigate this possibility.

## Electronic supplementary material

Dataset 1
