# Author Correction: Canonical germinant receptor is dispensable for spore germination in *Clostridium botulinum* group II strain NCTC 11219

**DOI:** 10.1038/s41598-020-60486-5

**Published:** 2020-03-04

**Authors:** Charlien Clauwers, Cédric Lood, Bram Van den Bergh, Vera van Noort, Chris W. Michiels

**Affiliations:** 1 Laboratory of Food Microbiology and Leuven Food Science and Nutrition Research Centre (LFoRCe), KU Leuven, Leuven, Belgium; 2 Centre of Microbial and Plant Genetics, KU Leuven, Leuven, Belgium

Correction to: *Scientific Reports* 10.1038/s41598-017-15839-y, published online 13 November 2017

Bram Van den Bergh was omitted from the author list in the original version of this Article. This has been corrected in the PDF and HTML versions of the Article, and in the accompanying Supplementary Information file.

The Author Contributions section now reads:

CC designed the experiments with CWM, executed the experiments, analyzed the data, and wrote the manuscript. CL and VvN conducted the in silico analysis of Ger receptor genes in 152 gIICb strains. BVdB performed the bioinformatic analysis of the genome sequence of the gerBAC deletion mutant. CWM conceived the study and finalized the manuscript. All authors discussed and interpreted the data.

