# Supplementary material for: Canonical germinant receptor is dispensable for spore germination in Clostridium botulinum group II strain NCTC 11219
Source: Sci Rep. 2017 Nov 13;7:15426. doi: 10.1038/s41598-017-15839-y (PMC5684421; doi:10.1038/s41598-017-15839-y)
Supplement: Supplementary file 1 — Dataset 1 [file 41598_2017_15839_MOESM1_ESM.docx]

**Canonical germinant receptor is dispensable for spore germination in *Clostridium botulinum* group II strain NCTC 11219**

Charlien Clauwers,^a^ Cédric Lood,^b^ Bram Van den Bergh^b^, Vera van Noort ^b^ and Chris W. Michiels^a^*

^a^ Laboratory of Food Microbiology and Leuven Food Science and Nutrition Research Centre (LFoRCe), and ^b^ Centre of Microbial and Plant Genetics, KU Leuven, Leuven, Belgium

*corresponding author: chris.michiels@kuleuven.be.

**Supplementary Materials**

Table S1: List of strains eliminated from the in silico analysis due to poor assembly performance. Strains were retrieved from the NCBI Sequence Read Archive database.

| Strain SRA ID | N50 |
| --- | --- |
| SRR2059456 | 5545 |
| SRR2059457 | 8125 |
| SRR2059458 | 7237 |
| SRR2059467 | 5580 |
| SRR2059478 | 3257 |
| SRR2059507 | 6370 |
| SRR2059516 | 4234 |
| SRR2059523 | 9721 |
| SRR2059531 | 8902 |
| SRR2059551 | 9157 |
| SRR2059553 | 3820 |
| SRR2059556 | 8623 |
| SRR2059561 | 7146 |
| SRR2059580 | 6816 |
| SRR2059591 | 4004 |
| SRR2059597 | 2997 |
| SRR2059599 | 5525 |

Table S2: In silico analysis of Ger receptor genes in 135 gIICb strains, retrieved from the NCBI Sequence Read Archive database. Percentage positive-scoring substitutions is shown as calculated by NCBI Blastp against the GerX3b subunits present in strain Eklund 17B.

|  | GerC (CLL_A3167) | GerA (CLL_A3168) | GerB (CLL_A3169) |
| --- | --- | --- | --- |
| SRR2059450 | 100 | 100 | 100 |
| SRR2059451 | 100 | 100 | 100 |
| SRR2059452 | 98.12 | 99.14 | 97.26 |
| SRR2059453 | 100 | 99.78 | 100 |
| SRR2059454 | 100 | 100 | 100 |
| SRR2059455 | 100 | 100 | 100 |
| SRR2059459 | 99.73 | 100 | 100 |
| SRR2059460 | 99.73 | 100 | 100 |
| SRR2059461 | 98.12 | 99.14 | 96.99 |
| SRR2059462 | 100 | 99.78 | 100 |
| SRR2059463 | 98.12 | 99.14 | 96.99 |
| SRR2059464 | 98.12 | 99.14 | 97.26 |
| SRR2059465 | 98.12 | 99.14 | 96.99 |
| SRR2059466 | 98.12 | 99.14 | 96.99 |
| SRR2059468 | 0 | 99.14 | 97.26 |
| SRR2059469 | 98.12 | 99.14 | 96.99 |
| SRR2059471 | 98.12 | 99.14 | 97.8 |
| SRR2059472 | 98.12 | 99.14 | 97.26 |
| SRR2059473 | 98.12 | 99.14 | 96.99 |
| SRR2059474 | 98.12 | 99.14 | 96.99 |
| SRR2059475 | 98.12 | 99.14 | 96.99 |
| SRR2059476 | 98.12 | 99.14 | 96.99 |
| SRR2059477 | 98.12 | 99.14 | 96.99 |
| SRR2059479 | 98.12 | 99.14 | 96.99 |
| SRR2059480 | 98.12 | 99.14 | 96.99 |
| SRR2059481 | 98.12 | 99.35 | 96.99 |
| SRR2059482 | 98.12 | 99.14 | 96.99 |
| SRR2059483 | 98.12 | 99.14 | 96.99 |
| SRR2059484 | 98.12 | 99.14 | 97.26 |
| SRR2059485 | 98.12 | 99.14 | 96.99 |
| SRR2059486 | 98.12 | 99.14 | 96.99 |
| SRR2059487 | 98.12 | 99.14 | 96.99 |
| SRR2059488 | 98.12 | 99.14 | 96.99 |
| SRR2059489 | 98.12 | 99.14 | 96.99 |
| SRR2059490 | 98.12 | 99.14 | 98.68 |
| SRR2059491 | 98.12 | 99.14 | 96.99 |
| SRR2059492 | 98.12 | 99.14 | 96.99 |
| SRR2059493 | 98.12 | 99.14 | 96.94 |
| SRR2059494 | 98.12 | 0 | 97.26 |
| SRR2059495 | 98.12 | 99.14 | 97.26 |
| SRR2059496 | 98.12 | 99.14 | 96.99 |
| SRR2059497 | 98.12 | 99.14 | 96.99 |
| SRR2059499 | 98.12 | 99.14 | 96.99 |
| SRR2059500 | 98.12 | 99.14 | 96.99 |
| SRR2059501 | 98.12 | 99.14 | 97.26 |
| SRR2059502 | 97.71 | 98.96 | 97.26 |
| SRR2059503 | 98.12 | 99.14 | 0 |
| SRR2059504 | 98.12 | 99.14 | 96.99 |
| SRR2059505 | 98.12 | 99.14 | 96.99 |
| SRR2059506 | 98.12 | 99.14 | 96.99 |
| SRR2059508 | 98.12 | 99.14 | 96.99 |
| SRR2059509 | 98.12 | 99.14 | 96.99 |
| SRR2059510 | 98.12 | 99.14 | 97.26 |
| SRR2059511 | 98.12 | 99.14 | 97.26 |
| SRR2059512 | 98.12 | 99.14 | 96.99 |
| SRR2059513 | 98.12 | 99.14 | 96.99 |
| SRR2059514 | 98.12 | 99.14 | 96.99 |
| SRR2059515 | 98.12 | 99.14 | 96.99 |
| SRR2059517 | 100 | 99.78 | 100 |
| SRR2059518 | 98.12 | 99.14 | 96.99 |
| SRR2059519 | 98.12 | 99.14 | 97.26 |
| SRR2059520 | 98.12 | 99.14 | 96.99 |
| SRR2059521 | 98.12 | 99.14 | 96.99 |
| SRR2059522 | 98.12 | 99.14 | 96.99 |
| SRR2059524 | 98.12 | 99.14 | 96.99 |
| SRR2059525 | 98.12 | 99.14 | 96.99 |
| SRR2059526 | 98.12 | 99.14 | 96.99 |
| SRR2059527 | 98.12 | 99.14 | 96.99 |
| SRR2059528 | 98.12 | 99.14 | 97.83 |
| SRR2059529 | 98.12 | 99.14 | 96.99 |
| SRR2059530 | 98.12 | 99.14 | 96.99 |
| SRR2059532 | 98.12 | 99.14 | 96.99 |
| SRR2059533 | 98.12 | 99.14 | 97.26 |
| SRR2059534 | 98.12 | 99.14 | 96.99 |
| SRR2059535 | 98.12 | 99.14 | 96.99 |
| SRR2059536 | 98.12 | 99.14 | 96.99 |
| SRR2059537 | 98.12 | 99.14 | 96.99 |
| SRR2059538 | 98.12 | 99.14 | 96.99 |
| SRR2059539 | 98.12 | 99.14 | 96.99 |
| SRR2059540 | 98.12 | 99.14 | 96.99 |
| SRR2059541 | 98.12 | 99.14 | 96.99 |
| SRR2059542 | 98.12 | 99.14 | 96.99 |
| SRR2059543 | 98.12 | 99.14 | 96.99 |
| SRR2059544 | 98.12 | 99.14 | 96.99 |
| SRR2059545 | 98.12 | 99.14 | 96.99 |
| SRR2059546 | 98.12 | 99.14 | 96.99 |
| SRR2059547 | 98.12 | 99.14 | 96.99 |
| SRR2059548 | 98.12 | 99.14 | 96.99 |
| SRR2059549 | 98.12 | 99.14 | 96.99 |
| SRR2059550 | 98.12 | 99.14 | 96.99 |
| SRR2059552 | 98.12 | 99.14 | 96.99 |
| SRR2059554 | 98.12 | 99.14 | 96.99 |
| SRR2059555 | 98.12 | 99.14 | 96.99 |
| SRR2059557 | 98.12 | 99.14 | 96.99 |
| SRR2059558 | 98.12 | 99.14 | 96.99 |
| SRR2059559 | 98.12 | 99.14 | 97.26 |
| SRR2059560 | 98.12 | 99.14 | 96.99 |
| SRR2059562 | 98.12 | 99.14 | 96.99 |
| SRR2059563 | 98.12 | 99.14 | 96.99 |
| SRR2059564 | 98.12 | 99.14 | 96.99 |
| SRR2059565 | 98.12 | 99.14 | 97.26 |
| SRR2059566 | 98.12 | 99.14 | 96.30 |
| SRR2059567 | 98.12 | 99.14 | 96.99 |
| SRR2059568 | 98.12 | 99.14 | 96.99 |
| SRR2059569 | 98.12 | 99.14 | 96.99 |
| SRR2059570 | 98.12 | 99.14 | 96.99 |
| SRR2059571 | 98.12 | 99.14 | 97.26 |
| SRR2059572 | 98.12 | 99.14 | 97.26 |
| SRR2059573 | 98.12 | 99.14 | 96.99 |
| SRR2059574 | 98.12 | 99.14 | 97.26 |
| SRR2059575 | 98.12 | 99.14 | 96.99 |
| SRR2059576 | 98.12 | 99.14 | 96.99 |
| SRR2059577 | 98.12 | 99.14 | 96.99 |
| SRR2059578 | 98.12 | 99.14 | 96.99 |
| SRR2059579 | 98.12 | 99.14 | 96.99 |
| SRR2059581 | 98.12 | 99.14 | 96.99 |
| SRR2059582 | 98.12 | 99.14 | 96.99 |
| SRR2059583 | 98.12 | 99.14 | 96.99 |
| SRR2059584 | 98.12 | 99.14 | 97.26 |
| SRR2059585 | 98.12 | 99.14 | 96.99 |
| SRR2059586 | 98.12 | 99.14 | 96.99 |
| SRR2059587 | 98.12 | 99.14 | 96.99 |
| SRR2059588 | 98.12 | 99.14 | 96.99 |
| SRR2059589 | 0 | 99.14 | 96.99 |
| SRR2059590 | 98.12 | 99.14 | 96.99 |
| SRR2059592 | 98.12 | 99.14 | 96.99 |
| SRR2059593 | 98.12 | 99.14 | 96.99 |
| SRR2059594 | 98.12 | 99.14 | 96.99 |
| SRR2059595 | 98.12 | 99.14 | 96.99 |
| SRR2059596 | 98.12 | 99.14 | 96.99 |
| SRR2059598 | 98.12 | 99.14 | 97.26 |
| SRR2059600 | 98.12 | 99.14 | 96.99 |
| SRR2059601 | 97.99 | 99.14 | 96.99 |
| SRR2059602 | 98.12 | 99.14 | 0 |
| SRR2059603 | 98.12 | 99.14 | 97.26 |

Note: the score in five cases shows to be zero. Close inspection of the assemblies in those cases revealed that the neighbouring gene or two genes are at the edge of a contig, and that the genes were probably missed by the assembly software due to low depth of coverage in that region. For the assembly of SRR2059494, for example, the gene GerA is missing, and both GerC and GerB can be found on the edges of 2 different contigs.
